# Potential of Cranberry Jelly to Prevent Urinary Stone Formation After Cutaneous Ureterostomy: A Case Report

**DOI:** 10.7759/cureus.54819

**Published:** 2024-02-24

**Authors:** Kanya Kaga, Mayuko Kaga

**Affiliations:** 1 Department of Urology, Chiba Prefectural Sawara Hospital, Chiba, JPN; 2 Department of Urology, Mihama Narita Clinic, Chiba, JPN

**Keywords:** magnesium ammonium phosphate, urinary stone, catheter, cutaneous ureterostomy, cranberry jelly

## Abstract

One complication of cutaneous ureterostomy is urinary stone formation, which may lead to recurrent pyelonephritis. Frequent catheter changes and the prophylactic administration of antibiotics are commonly used to prevent stone formation. Cranberry products have been reported to inhibit stone formation in indwelling urethral catheters. We herein examined the inhibitory effects of a cranberry product on stone formation in a case of catheter occlusion due to stone formation after cutaneous ureterostomy. The results obtained indicate the potential of cranberry products to prevent stone formation after cutaneous ureterostomy requiring catheter placement.

## Introduction

Cutaneous ureterostomy is the least invasive technique for urinary diversion [1]. Although there are tubeless techniques, a catheter is placed in the ureter for periodic replacement to prevent blockage of the fistula in some cases. Catheter replacement was required in 26% of cutaneous ureterostomy after radical cystectomy, which was not a small proportion [2]. Catheter-related complications have been reported. Catheter obstruction due to stone adhesion during catheter replacement is a common complication that may lead to recurrent pyelonephritis, which requires treatment with the prophylactic administration of antibiotics and frequent catheter replacement [3]. Cranberry products have been reported to inhibit the adhesion of stones to indwelling urethral catheters and, thus, prevent obstruction [4-5]. We herein report the effects of the administration of a cranberry product in a case of cutaneous ureterostomy with repeated catheter occlusion.

## Case presentation

**Figure 1:**
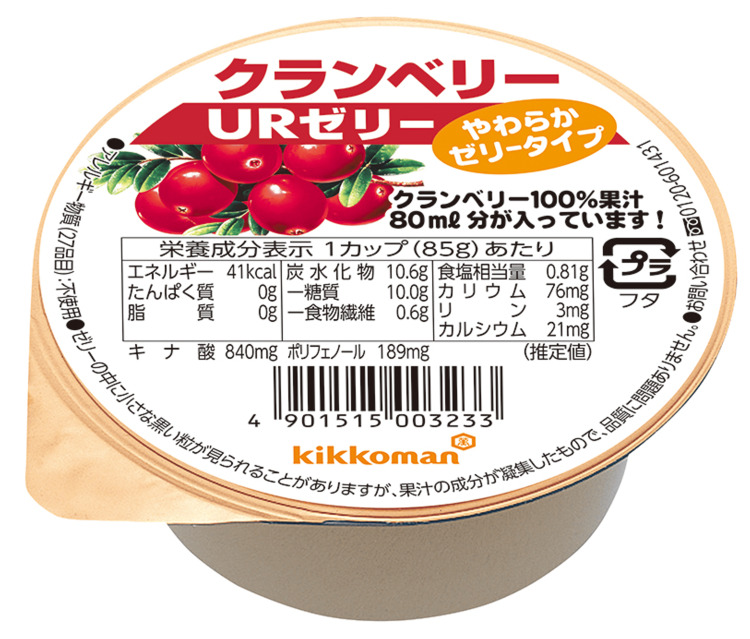
Kikkoman cranberry UR jelly The front of the Kikkoman cranberry UR jelly package states the food ingredients in Japanese. It contains 80 mL of 100% cranberry juice. The content is 85 g per cup. Nutritional ingredients include 41 calories of energy, 0 g of protein, 0 g of fat, 10.6 g of carbohydrate (10.0 g of sugar and 0.6 g of dietary fiber), 0.81 g of salt, 76 mg of potassium, 3 mg of phosphorus, 21 mg of calcium, 840 mg of quinic acid, and 189 mg of polyphenols.

An 82-year-old Japanese man underwent total gastrectomy and low anterior resection for gastric and rectal cancer. The left ureteral injury occurred intraoperatively; therefore, a cutaneous ureterostomy was performed at the Urology Department of another hospital. He was referred to our department for periodic ureteral catheter replacement. He had post-operative gastric and rectal cancer, as well as diabetes and dyslipidemia. The catheter was changed regularly every six weeks, and a blockage at the tip of the pelvic side of the catheter was noted each time. A stone analysis revealed magnesium ammonium phosphate in the obstruction, indicating that the cause of the obstruction was stone formation. The catheter used was made of polyurethane and was the Urostent System (Create Medic CO., LTD., Yokohama, Japan). Shortening the time of catheter replacement was considered; however, the patient refused. Since cranberry products have been shown to effectively prevent the obstruction of indwelling urinary catheters, we examined the efficacy of a cranberry product administered for 24 weeks to prevent catheter obstruction. The protocol was approved by the Institutional Review Board of Chiba Prefectural Sawara Hospital (approval number: 724-5-6). The patient provided informed consent before the initiation of treatment. Due to the risk of aspiration, 85 g of cranberry jelly containing 80 mL of 100% cranberry juice, Kikkoman cranberry UR jelly (Kikkoman Nutricare Japan Inc., Tokyo, Japan), was used (Figure 1).

**Figure 2:**
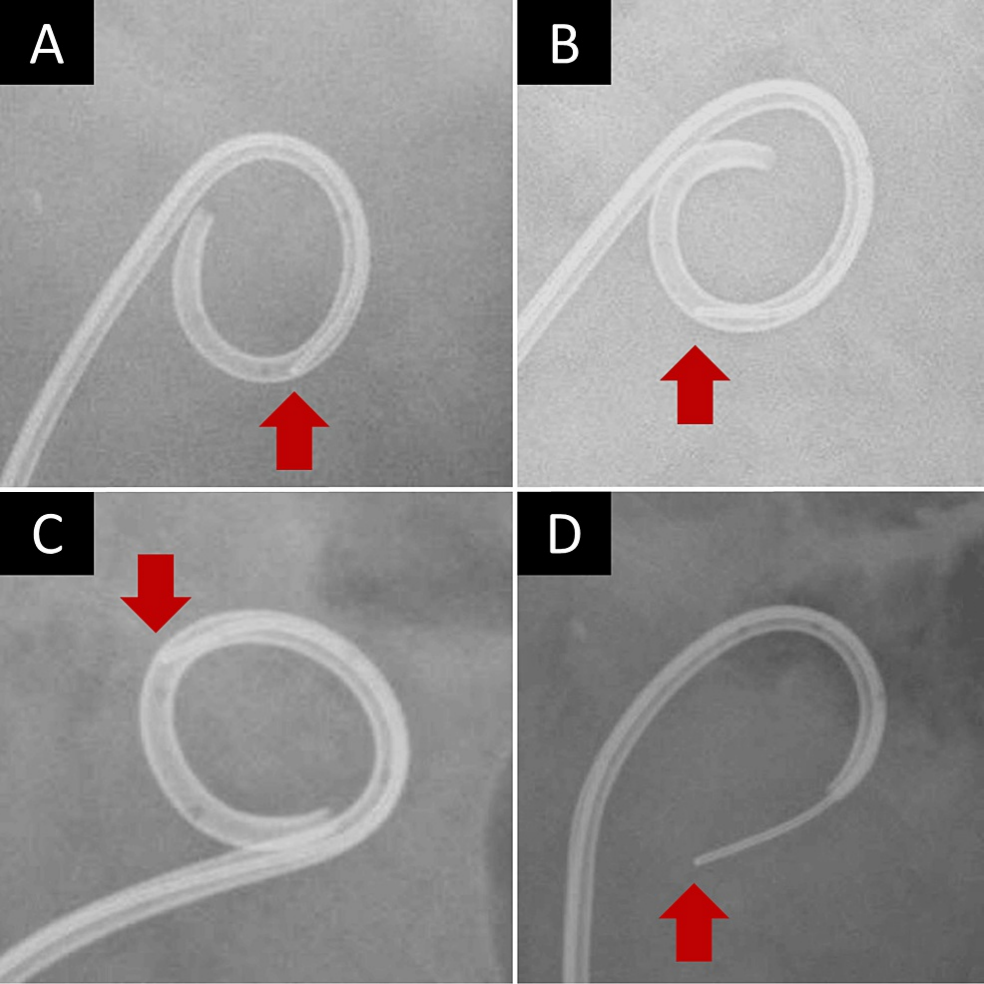
Fluoroscopic findings of catheter replacement after cutaneous ureterostomy (A) 0 weeks (before cranberry jelly intake). (B) Six weeks after intake of cranberry jelly. (C) 12 weeks after intake of cranberry jelly. (D) 18 weeks after intake of cranberry jelly The image shows the old catheter at the time of replacement. Red arrow: the tip of the guidewire

Cranberry jelly was provided directly to the patient by Kikkoman Nutricare Japan Inc. The patient consumed cranberry jelly once a day. Blockage of the catheter tip was no longer observed 18 weeks after the initiation of treatment, and there was no blockage after 24 weeks of treatment (Figure 2).

**Figure 3:**
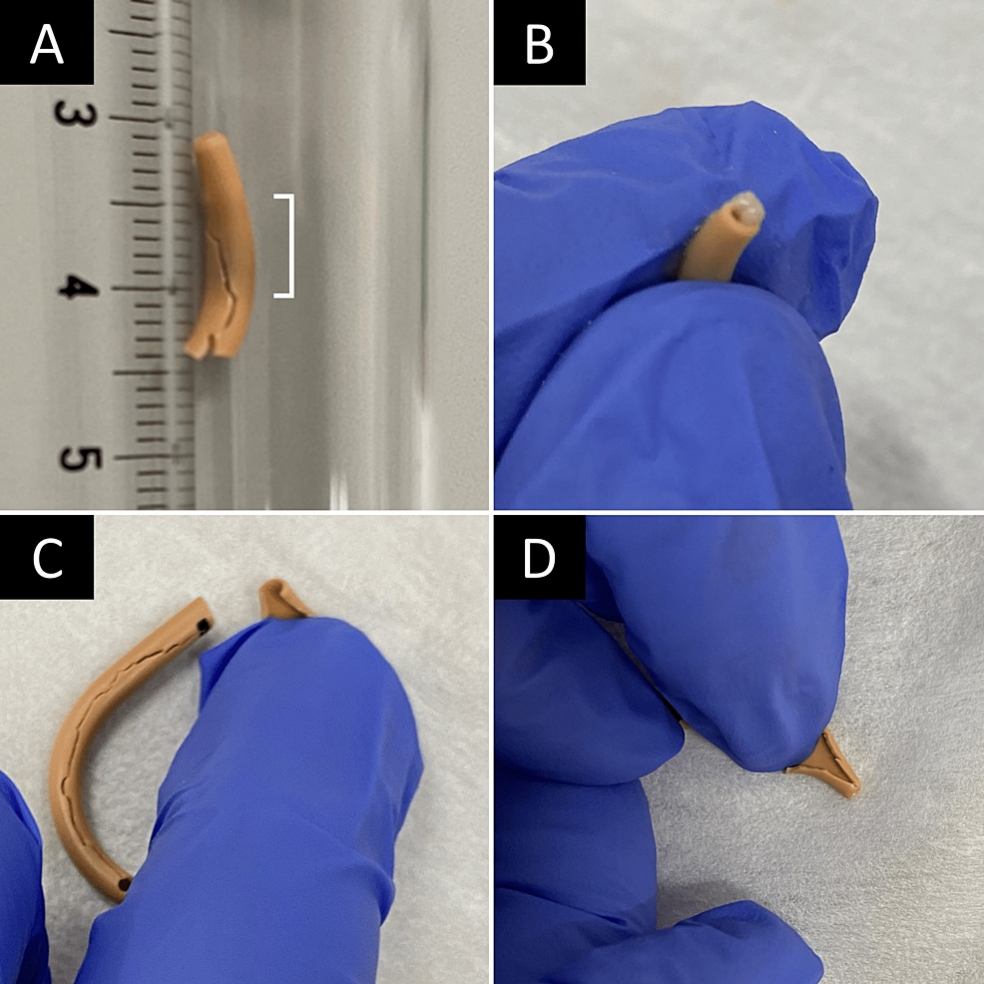
Status of stones adhering to catheters (A) 0 weeks (before cranberry jelly intake). (B) 12 weeks after the intake of cranberry jelly. (C) 18 weeks after the intake of cranberry jelly. (D) 24 weeks after the intake of cranberry jelly Right square bracket at 0 weeks: area blocked by adhering stones. The stones were adherent to an area 1 cm from the tip of the catheter.

Milky white stones were present in the obstruction in the early stages of treatment but were not detected after 18 weeks of treatment (Figure 3).

Renal pelvis urine pH was 6.5 before the start of treatment but was alkalized to pH 7.0 after 12 weeks of treatment and pH 7.5 after 24 weeks of treatment. The number of leukemic cells in the urine remained unchanged after the stones were no longer adherent. The patient has since requested to continue taking cranberry jelly, and the catheter has been replaced without blockage.

## Discussion

Previous studies demonstrated the utility of cranberry products in the prevention of urinary tract infections and the management of peristomal skin [6-8]. Aoki et al. reported that cranberry juice was effective in the treatment of indwelling urethral catheter obstruction due to magnesium ammonium phosphate stones. Although cranberry products effectively prevented the obstruction of indwelling urethral catheters in Japan, Morris and Stickler reported that they were ineffective; therefore, the effects of cranberry products on catheter blockage are controversial [4-5,9-10]. This discrepancy may be attributed to racial differences.

To the best of our knowledge, the effects of cranberry products on the occlusion of catheters after cutaneous ureterostomy have not yet been examined. In the present case, cranberry jelly was effective against catheter occlusion after cutaneous ureterostomy. Regarding indwelling urinary catheter obstruction, cranberry products administered for three to six months were shown to reduce the adhesion of stones to the catheter [4]. Consistent with these findings, catheter obstruction improved in the present case after 18 weeks of treatment.

Regarding the mechanisms by which stone formation is inhibited, quinic acid in cranberry products has been shown to acidify urine and prevent the precipitation of magnesium-ammonium phosphate; however, this mechanism was ruled out in the present case because renal pelvis urine was alkalized during the treatment period [11]. Furthermore, a decrease in the number of leukemic cells in urine was found to reduce the substrate to which phosphate crystals adhere, thereby preventing the formation of urinary stones [12]. This mechanism was not applicable in the present case because the number of leukemic cells remained unchanged after 18 weeks of treatment when adherent stones had disappeared. Previous studies reported that cranberry products exerted anti-biofilm effects [13-14]. Proanthocyanidins in cranberries were shown to exert anti-adhesive effects against bacteria, suggesting that they inhibit biofilm formation and reduce stone adhesion [15].

A limitation of the present study was that the patient was instructed to continue his normal life, except for the consumption of cranberry jelly, and his living conditions during the observation period were not considered. In addition, since this was a case report, further validation is needed with a larger number of patients. However, the result showed the potential of cranberry jelly to prevent urinary stone formation after cutaneous ureterostomy.

The prevention of catheter blockage by the consumption of cranberry products, as in the present case, may prevent frequent catheter replacements and contribute to reductions in healthcare costs.

## Conclusions

In the present study, a cranberry product was useful for preventing catheter obstruction after cutaneous ureterostomy. It was suggested that this may be a new option for the prevention of catheter obstruction after cutaneous ureterostomy. As this was a case report, further validation is needed.
